# Intravenous Lipid Emulsions Affect Respiratory Outcome in Preterm Newborn: A Case-Control Study

**DOI:** 10.3390/nu13041243

**Published:** 2021-04-09

**Authors:** Giovanni Boscarino, Maria Giulia Conti, Francesca De Luca, Maria Di Chiara, Giorgia Deli, Marco Bianchi, Paola Favata, Viviana Cardilli, Giovanni Di Nardo, Pasquale Parisi, Gianluca Terrin

**Affiliations:** 1 Department of Maternal and Child Health, Policlinico Umberto I Hospital, Sapienza University of Rome, 00161 Rome, Italy; giovanni.boscarino@yahoo.com (G.B.); mariagiulia.conti@uniroma1.it (M.G.C.); francesca27deluca@gmail.com (F.D.L.); maria.dichiara@uniroma1.it (M.D.C.); giorgia.deli7@gmail.com (G.D.); marbi1996@gmail.com (M.B.); favata.paola@alice.it (P.F.); viviana.cardilli@uniroma1.it (V.C.); 2 Department of Molecular Medicine, Sapienza University of Rome, 00185 Rome, Italy; 3 Department of Pediatrics, Mental Health & Sense Organs (NESMOS), Faculty of Medicine & Psychology, c/o Sant’ Andrea Hospital, Sapienza University, 00189 Rome, Italy; giovanni.dinardo@uniroma1.it (G.D.N.); pasquale.parisi@uniroma1.it (P.P.)

**Keywords:** hypertriglyceridemia, parenteral nutrition, bronchopulmonary dysplasia (BPD), invasive mechanical ventilation, respiratory distress syndrome, mortality

## Abstract

(1) Background: Hypertriglyceridemia (HiTG) is a metabolic complication of intravenous lipid emulsions (ILEs) infusion. We aimed to evaluate the influence of HiTG on the respiratory outcome of preterm babies; (2) Methods: We enrolled, in a case–control study, newborns with gestational age <32 weeks or birth weight <1500 g, over a 3-year period. They were divided into cases and controls; cases were defined by the detection of HiTG defined as serum triglycerides (TG) value >150 mg/dL; (3) Results: We enrolled 40 cases and 105 controls. Cases had an increased incidence of bronchopulmonary dysplasia (30.0% vs. 14.3%, *p* < 0.05) and longer duration of invasive mechanical ventilation (7 days, 95% CI 4–10 days vs. 4 days, 95% CI 1–7 days, *p* < 0.01) compared to controls. Multivariate analysis confirmed that HiTG independently influenced the duration of invasive mechanical ventilation, also in the subgroups with gestational age ≤28 + 6/7 weeks or birth weight ≤1000 g; (4) Conclusion: Newborns with HiTG related to ILEs had a longer duration of invasive mechanical ventilation. Temporary suspension or reduction in ILEs in the case of HiTG is associated with an improvement of respiratory outcome.

## 1. Introduction

Malnutrition is one of the major prematurity-related complications. To reduce extra-uterine growth retardation (EUGR) as a consequence of malnutrition, current European Society of Pediatric Gastroenterology, Hepatology and Nutrition (ESPGHAN) guidelines recommend high energy intake in parenteral nutrition (PN) soon after birth [1]. This approach is referred to as “aggressive” nutrition. Intravenous lipid emulsions (ILEs), used as part of the total PN, are essential to meet the current nutritional recommendations for preterm newborns [2]. Despite the fact that appropriate energy intake is a primary goal of neonatal nutrition, the safety of high doses of lipids administered by PN is still debated [3,4,5,6]. High serum levels of triglycerides (TG) are recognized as a metabolic complication of ILEs infusion, especially in very low birth weight infants (VLBW) [2]. In this population, the risk of hypertriglyceridemia (HiTG) is greater because of limited muscle and fat mass and reduced metabolic capacity due to a decreased hydrolytic capacity of the enzyme lipoprotein lipase [7]. The administration of ILEs has been associated with worse respiratory function in adult patients. This aspect is still controversial in the newborns. Early administration of ILEs has been associated with an increased pulmonary vascular resistance [8] and pulmonary arterial lipid deposits have been detected in histological findings of human neonates receiving ILEs [9]. However, less recent evidence did not confirm this association [10]. Thus, the clinical consequences of HiTG on respiratory outcome remain to be defined.

Starting from these considerations, we designed a study to evaluate the relation between HiTG and mechanical ventilation support in preterm newborns.

## 2. Materials and Methods

### 2.1. Study Design and Population

Newborns with gestational age (GA) <32 weeks or birth weight (BW) <1500 g, receiving PN and consecutively admitted to the neonatal intensive care unit (NICU) of Policlinico Umberto I Hospital, from January 2017 to November 2019 were enrolled in our study. Newborns with congenital diseases, inborn errors of metabolism, congenital infections, familiar history of allergy, use of pre- or probiotics during hospital stay and hospital discharge or death within 72 h of life were excluded [11,12,13,14,15,16]. Our population was divided in two study groups, according with plasma TG values: cases (newborns with at least one measurement of serum TG >150 mg/dL associated with ILEs) and controls (newborns with serum TG never exceeding 150 mg/dL). In our preliminary data, we observed a relation between respiratory condition and levels of TG >150 mg/dL. Thus, we used in this study the threshold of 150 mg/dL to define the condition of HiTG. Our primary outcome was the rate of prolonged invasive mechanical ventilation (more the 7 days) while our secondary outcome was the rate of mortality during hospital stay.

### 2.2. Data Collection

Prenatal, perinatal, and postnatal data were retrospectively recorded in a specific data form by researchers not involved in routine clinical practice. In our policy, we collected nutritional data daily on PN, EN and feeding tolerance in a specific database [3,5].

In addition, data regarding neonatal morbidities and administration of invasive or noninvasive mechanical ventilation were collected. We defined morbidity as the presence of at least one of the major prematurity complications including necrotizing enterocolitis (NEC) Bell-Stage ≥II, periventricular leukomalacia (PVL), late-onset culture proven sepsis, retinopathy of prematurity (ROP) and bronchopulmonary dysplasia (BPD). The diagnoses of neonatal morbidities were performed according with standard criteria, by physicians unaware of the study design and aims, as previously described [17,18]. We defined “improved respiratory outcome” as the passage from invasive to non-invasive mechanical ventilation or not free oxygen and/or reduction of oxygen requirement. The extrauterine growth restriction (EUGR) was defined in the longitudinal way if the weight loss was more than one SD between birth and discharge [19]. A blinded statistician performed data analysis.

### 2.3. Nutritional Protocol

Nutritional (enteral and parenteral) protocol was previously described [3,20]. In brief, the mother’s own milk was administered as soon as possible after birth [20]. Preterm formula was administered when human milk was not available or sufficient. Donor human milk was not available during the entire study period. Minimal enteral feeding (MEF) was started at 10–20 mL/kg/day. The amount was increased by 20–30 mL/kg/day if enteral nutrition was tolerated [21,22].

The PN, started early after birth, was administered via a central venous access device to maintain adequate fluids, electrolytes and nutrient intakes until full enteral feeding (FEF, 120 kcal/kg/day) was achieved [3]. As previously described [23], initial lipid (Smoflipid ^®^; Fresenius Kabi, Lake Zurich, IL, USA) intake was 1 g/kg/day, progressively increased by 0.5–1 g/kg up to 3.5 g/kg/day at 7 days of life (DOL). We defined total PN (TPN) when PN represented more than 70% of total nutrition (enteral and parenteral) during the first 7 DOL. Plasma TG were measured every 72 h for all enrolled neonates in PN with ILEs infusion.

### 2.4. Statistics

Statistical analysis was performed using Statistical Package for Social Science software (SPSS Inc., Chicago, IL, USA), version 25.0. We checked for normality using Shapiro–Wilk test. The mean and 95% confidence interval (CI) summarized continuous variables and number and percentage described categorial variables. We used χ^2^ test for categorical variable and t-test or Mann–Whitney for paired and unpaired variables.

To evaluate the influence of HiTG on respiratory outcome, independently to GA or BW, we identified a subgroup of newborns with GA ≤28 + 6/7 weeks or BW ≤1000 g (extremely low birth weight, ELBW). We performed a binary regression analysis to evaluate the influences of covariates (FEF reached after 15 DOL, HiTG, administration of prenatal steroids prophylaxis and surfactant and to be small for gestational age, small for gestational age (SGA) at birth) on the duration of prolonged invasive mechanical ventilation (more than 7 days), on BPD and on post-natal steroids administration for the overall population and for the subgroup with GA ≤28 + 6/7 weeks or ELBW. The level of significance for all statistical tests was 2-sided (*p* < 0.05). In addition, we performed a binary regression analysis to evaluate the influence of covariates (HiTG, gender, to be SGA at birth, Apgar score ≥ 5, pH on cord blood ≥ 7.1 and morbidity) on secondary outcome.

## 3. Results

**Figure 1:**
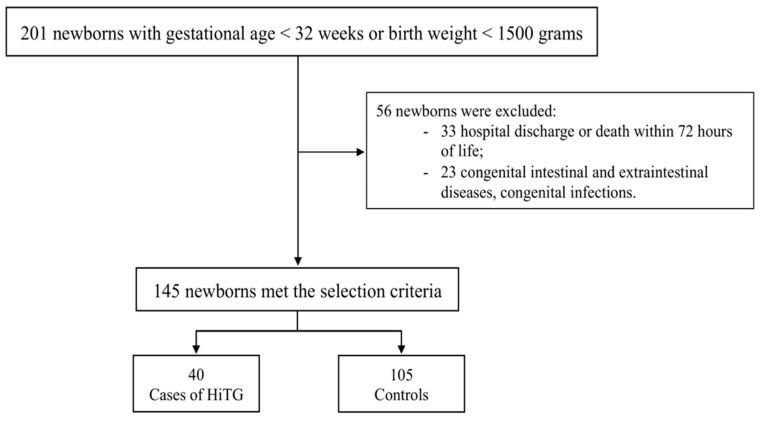
Flow-chart. Notes. HiTG (hypertriglyceridemia).

Of the 201 eligible newborns, 145 met the selection criteria (Figure 1).

Data regarding baseline clinical characteristics were reported in Table 1. Newborns in the cases group had lower GA and BW and were more SGA at birth compared to newborns in the controls group (Table 1).

Cases (102 days, 95% CI 70 to 134 days) had a longer length of hospital stay compared to controls (87 days, 95% CI 72 to 102 days, *p* = 0.005). The percentage of newborns fed by enteral nutrition in the first 72 h of life was lower in cases (50%) compared to controls (87.6%, *p* < 0.001); cases achieved FEF later in life (36 days, 95% CI 23 to 48 days) in comparison with controls (21 days, 95% CI 17 to 25 days, *p* < 0.001). The percentage of neonates receiving TPN was similar between the two groups (cases 75.0% vs. controls 66.7%, *p* = 0.057). The rate of morbidity and mortality were similar between cases and controls (40.0% vs. 28.6%, *p* = 0.080; 12.5% vs. 3.8%, *p* = 0.052, respectively).

Cases showed a higher rate of newborns with prolonged mechanical ventilation (Table 2). We also observed a longer time of invasive mechanical ventilation, higher incidence of BPD and higher rate of newborns receiving post-natal steroids compared with controls (Table 2).

**Figure 2:**
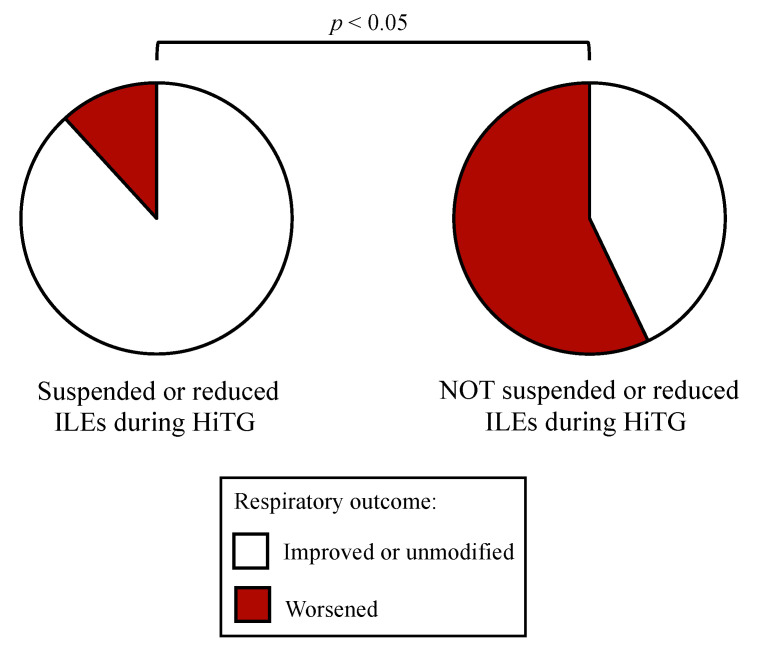
Respiratory outcome of newborns with respiratory distress syndrome during parenteral nutrition infusion before and after a hypertriglyceridemia episode. Notes. ILEs (intravenous lipid emulsions); HiTG (hypertriglyceridemia).

Newborns with respiratory distress syndrome and HiTG worsened the respiratory outcome, when the infusion of ILEs was not suspended or reduced, more frequently (57.1%) compared to newborns that suspended or reduced the infusion of ILEs, (11.8% *p* = 0.038) (Figure 2).

Newborns with HiTG for whom ILEs were suspended or reduced did not show differences on energy (762.5 kcal/kg/week 95% CI 578.9 to 946.1 kcal/kg/week vs. 714.9 kcal/kg/week 95% CI 571.2 to 858.7 kcal/kg/week, *p* = 0.930) and lipids (24.3 g/kg/week 95% CI 18.1 to 30.5 g/kg/week vs. 22.7 g/kg/week 95% CI 18.4 to 27.0 g/kg/week, *p* = 1000) intakes given by PN in the first 7 DOL, compared to neonates for whom ILEs were not suspended or reduced. Additionally, the rate of EUGR were similar between these groups of newborns (71.4% vs. 72.7%, *p* = 0.676).

Binary logistic regression analysis showed that HiTG is independently related with more than 7 days of invasive mechanical ventilation (Table 3). The multivariate analysis showed that BPD and post-natal steroids administration were not influenced by HiTG (OR 95% CI 2380, 0.769 to 7369, *p* = 0.133 and OR 95% CI 2643, 0.950 to 7350, *p* = 0.063, respectively).

The baseline clinical characteristics of newborns with GA ≤ 28 + 6/7 weeks or ELBW were showed in Table S1. Cases, in this subgroup, showed a higher rate and longer duration of invasive mechanical ventilation and more frequent use of post-natal steroids compared to controls (Table 2). In a sensitivity binary regression analysis, including only newborns of the subgroup, we observed that prolonged mechanical ventilation is independently related with HiTG (Table 3). In sensitivity multivariate analysis, BPD and post-natal steroids administration were not dependent on HiTG (OR 95% CI 2283, 0.650 to 8011, *p* = 0.193 and OR 95% CI 2441, 0.785 to 7587, *p* = 0.123, respectively). Mortality, for all newborns enrolled including those with GA ≤28 + 6/7 weeks or ELBW, appeared to not be influenced by covariates.

## 4. Discussion

We demonstrated that HiTG in the first 7 DOL is associated with a worse respiratory outcome in preterm newborns. In particular, we observed a higher risk of prolonged invasive mechanical ventilation related with HiTG. These findings are particularly evident in newborns with GA less than 28 weeks or body weight less than 1000 g at birth. A temporary suspension or reduction in ILEs rapidly improve the respiratory outcome in preterm newborns with HiTG. Finally, the occurrence of HiTG in babies born preterm did not influence the rate of mortality at discharge but prolonged the length of hospital stay.

Previous evidences suggest that higher plasma lipid concentrations could affect lung function in clinical settings different from the neonatal population [24,25,26]. In particular, an impairment of lung function, oxygenation, compliance of respiratory system, pulmonary vascular resistance and gas exchange following intravenous lipid infusion has been described in critically ill adults [24,25,26].

Few studies have examined this specific topic with regard to the neonatal population. Periera et al. [27] in a small study including 18 preterm newborns (BW ranging from 770–1890 g), evaluated the effects of ILEs on pulmonary function of premature infants divided in two groups according to postnatal age (<1 week vs. 2–3 weeks). The authors found that newborns with age <1 week developed a significant decrease in pO2 levels after 4 h from fat infusion. However, clinical outcomes such as the duration of mechanical ventilation or occurrence of BPD were not evaluated in this study. In a small study including 11 newborns with BWs less than 2000 g, AGA, who required respiratory support, Prasertsom et al. [8] observed a worsening of pulmonary vascular resistance associated with ILEs, and a recovery of baseline values after 24 h from ILEs discontinuation. Additionally, in this paper, no data were reported regarding the effect of ILEs or HiTG on clinical outcome. More recently, in a retrospective study, Giretti et al. [28] identified respiratory distress syndrome as a risk factor for HiTG in preterm newborns with BWs less than 1250 g. Despite the authors demonstrating an association between respiratory distress syndrome and lipid tolerance, the occurrence of respiratory distress syndrome preceded HiTG. In a similar population, the same research group, in a retrospective case–control study [29], did not find any relation between HiTG and BPD. Differently from our study, the authors did not correct the results for possible confounding variables. We found a higher occurrence of BDP and post-natal steroids administration in the case of HiTG, however, this relation was not confirmed by the multivariate models. The occurrence of BPD could be related to other factors not considered in the multivariate model and studies with more confounding variables could show a relation. Other well-conducted studies are needed to demonstrate a possible correlation. We considered as the primary outcome the rate of prolonged invasive mechanical ventilation (more the 7 days) and not the occurrence of BPD. We found a relation between HiTG and worsening respiratory outcome in a univariate analysis and we confirmed this relation in a multivariate model. In addition, we performed a sensitivity analysis in a subgroup of newborns with GA ≤28 + 6/ 7 weeks or ELBW, confirming the results observed in the entire study population.

Similar to previous studies, our findings suggest that GA, BW and to be SGA at birth were significant risk factors for the development of HiTG [28,30]. Similar to Sinclair et al. [30], the rate of mortality is not influenced by HiTG. However, Holtrop et al. [31] demonstrated that HiTG could influence the rate of mortality. This could be due to a different TG threshold and different sample size. Our multivariate analysis showed that mortality is not influenced by considered confounding variables (HiTG, gender, to be SGA at birth, Apgar score ≥5, pH on cord blood ≥7.1 and morbidity). Although we observed interesting results on the relation between mortality and HiTG, this study was not powered to demonstrate the relation between higher serum TG and the risk of exitus.

The appropriate threshold for serum TG in preterm infants is still a debated topic [30]. The incidence of HiTG and clinical consequences may vary among studies due to different TG thresholds, variable patient demographics and different infusion rates of ILEs [30]. According to the current guidelines for infants on PN, serum plasma TG should not exceed 265 mg/dL [2,31,32]. The differences with our results might be due also to a different threshold of serum TG levels. In our preliminary observation, we found a relation between respiratory conditions and levels of TG >150 mg/dL. Thus, we considered a threshold value of 150 mg/dL to define a case of HiTG. We confirmed significant respiratory effect associated with HiTG defined by this threshold. Despite further studies being advocated to determine the threshold value of serum TG as a maker of ILEs toxicity, on the bases of our results, we suggest considering that values more than 150 mg/dL of TG may have just an impact on respiratory outcome. Thus, we believe that the adjustment of PN prescription could be considered in newborn with respiratory distress and TG levels in the serum >150 mg/dL. It is important to monitor the metabolic status of infants in PN, in order to suspend or reduce the ILEs in case of HiTG. A well-designed large clinical trial is required to evaluate the effect of regular TG monitoring and titration of ILEs intake on respiratory clinical outcome. A randomized control trial (RCT) assessing the effects of ILEs related HiTG on respiratory clinical outcome in preterm infants is needed to confirm the results of our study. The ILEs infusion’s suspension is not recommended by actual guidelines in the case of HiTG (>265 mg/dL); instead, a temporary reduction in ILEs infusion assuring a minimum linoleic acid intake of 0.25 g/kg/day is recommended in preterm infants [2,33]. Despite the fact that reducing ILEs infusion when serum TG levels exceed 150 mg/dL may potentially compromise nutritional intakes in preterm infants dependent on PN [2], we did not find differences in energy intakes between neonates for whom ILEs were suspended or reduced and neonates for whom they were not. In addition, we also observed a similar occurrence rate of EUGR between these groups of neonates.

It has been reported that ILEs containing fish oil reduce the serum TG levels [34] and improve the respiratory outcome of preterm babies in PN [35]. Both our study groups received the same ILEs containing fish oil. Thus, we are not able to define the role of fish oil on respiratory outcome.

We hypothesized some mechanisms that may support our results. Preterm newborns in PN with ILEs infusion are at risk of developing lipid micro-emboli, with a decrease in diffusion capacity and oxygenation [9,25,27]. We speculate that this alteration in diffusion capacity unbalances the vasomotor tone, causing an alteration of the hemodynamic status of the lungs in this critical population. Increases in vascular tone and subsequent increases in pulmonary artery pressure may have an impact on respiratory distress syndrome. Vasudevan et al. [36] hypothesized that pulmonary artery pressure and eicosanoid metabolites are affected by ILEs in preterm babies. On the other hand, inflammatory cells and type II secretory phospholipase A possibly play a role in deteriorating lung injury [24]. Neutrophils or macrophages activated by lipids in the intracapillary or alveolar space release enzymes and inflammatory mediators, such as type II secretory phospholipase A and platelet activating factor [24]. These probably enhance the alveolar–capillary membrane permeability, tissue inflammation, and surfactant damage [24].

Despite being interesting, the results of our study should be interpreted taking into account several limitations. The association between worse respiratory outcome and HiTG may be related to the effects of chance (random error), bias or confounding factors. We verified that the effects on the lungs of HiTG persisted even after we corrected results for confounding variables for overall and newborn born before to 29 weeks of GA or ELBW at birth. However, confounding variables still unknown or not considered in our statistical analysis may have influenced the results. To limit observer bias, the data for the analysis were collected by researchers not involved in the eligibility assessment and who were unaware of the study outcome and design. We discussed and defined a protocol for the collection, measurement and interpretation of data before starting the study. Besides, a blinded statistician performed the data analysis.

## 5. Conclusions

The HiTG related to ILEs influence the respiratory outcome of babies born preterm, particularly when GA is ≤28 + 6/7 weeks or they are ELBW at birth. Levels of TG should be strictly monitored and the withdrawal or avoidance of ILEs should be considered in newborns with respiratory distress. Our results suggest monitoring the metabolic status of infants in PN, in order to suspend or reduce the ILEs in case of HiTG, thus improving the respiratory outcome of this critically ill population.

## Figures and Tables

**Table 1:** Baseline clinical characteristics of neonates with hypertriglyceridemia compared with controls.

	Cases of HiTG (*n* = 40)	Controls (*n* = 105)	OR (95% CI)	*p* Value
Maternal age, years	34 (29 to 38)	37 (34 to 40)	-	0.385
≥35 years, No. (%)	20 (50.0)	52 (49.5)	1.291 (0.587 to 2841)	0.525
Gestational age, weeks	26 (25 to 28)	28 (27 to 29)	-	**<0.001**
≤28 + 6/7 weeks, No. (%)	28 (70.0)	51 (48.6)	0.371 (0.167 to 0.822)	**0.013**
Birth weight, g	807 (633 to 981)	1066 (906 to 1226)	-	**<0.001**
ELBW, No. (%)	27 (67.5)	33 (31.4)	4909 (2217 to 10.872)	**<0.001**
SGA, No. (%)	12 (30.0)	17 (16.2)	2.701 (1132 to 6446)	**0.022**
Male sex, No. (%)	16 (40.0)	50 (47.6)	0.733 (0.350 to 1536)	0.410
Cesarean section, No. (%)	29 (72.5)	94 (89.5)	0.485 (0.172 to 1365)	0.136
Prenatal steroids administration ^a^, No. (%)	19 (47.5)	71 (67.6)	0.535 (0.247 to 1158)	0.110
Intrauterine growth restriction, No (%)	5 (12.5)	8 (7.6)	1956 (0.596 to 6417)	0.210
Gestational diabetes, No (%)	1 (2.5)	10 (9.5)	0.266 (0.033 to 2153)	0.168
Abruptio placentae, No (%)	4 (10.0)	10 (9.5)	1188 (0.348 to 4050)	0.501
Pregnancy-induced hypertension, No. (%)	11 (27.5)	26 (24.5)	1337 (0.579 to 3085)	0.495
Thyroid dysfunction, No. (%)	6 (15.0)	11 (10.5)	1.709 (0.583 to 5014)	0.240
Twins, No. (%)	10 (25.0)	36 (34.3)	0.737 (0.320 to 1696)	0.472
5-min Apgar score	6 (5 to 7)	7 (6 to 8)	-	0.078
<5, No. (%)	7 (17.5)	5 (4.8)	0.207 (0.061 to 0.702)	**0.012**
pH at birth	7.2 (7.1 to 7.3)	7.2 (7.1 to 7.3)	-	0.058
≤7.1, No. (%)	1 (2.5)	4 (3.8)	1414 (0.153 to 13.085)	0.614
Birth weight gain before 14 DOL, No. (%)	23 (57.5)	79 (75.2)	0.556 (0.234 to 1320)	0.180

**Notes.**^a^ Intramuscular steroid cycle in two doses of 12 mg over a 24-h period; HiTG (hypertriglyceridemia); ELBW (extremely low birth weight); SGA (small for gestational age); DOL (days of life). Data were expressed as mean (lower to upper limits 95% confidence interval), when not specified.

**Table 2:** Respiratory outcome of neonates with hypertriglyceridemia compared with controls.

	Overall		GA ≤ 28 + 6/7 Weeks or ELBW	
	Cases of HiTG (*n* = 40)	Controls (*n* = 105)	Cases of HiTG (*n* = 34)	Controls (*n* = 57)
Bronchopulmonary Dysplasia	12 (30.0) *	15 (14.3)	12 (35.3)	13 (22.8)
Caffeine administration	35 (87.5)	97 (92.4)	29 (85.3)	56 (98.2)
Postnatal steroids administration	13 (32.5) **	14 (13.3)	13 (38.2) *	12 (21.1)
Invasive mechanical ventilation	23 (57.5) **	34 (32.4)	22 (64.7) *	26 (45.6)
Duration, mean days (95% CI)	7 (4 to 10) **	4 (1 to 7)	7 (4 to 10) *	5 (1 to 8)
≥7 days	10 (25.0) **	6 (5.7)	10 (29.4) **	6 (10.5)
Noninvasive mechanical ventilation	34 (85.0)	89 (84.8)	29 (85.3)	49 (86.0)
Duration, mean days (95% CI)	16 (8 to 23)	16 (10 to 21)	16 (8 to 24)	18 (12 to 25)
≥7 days	16 (40.0)	29 (27.6)	16 (47.1)	26 (45.6)

**Notes.** GA (gestational age); ELBW (extremely low birth weight); * vs. controls *p* value < 0.05; ** vs. controls *p* value < 0.01. Data were expressed as No. (%), when not specified.

**Table 3:** Binary logistic regression analysis evaluating the influences of covariates on prolonged invasive mechanical ventilation (more than 7 days).

Covariates	Overall	GA ≤ 28 +6/7 Weeks or ELBW
FEF ≥ 15 days (not or yes)	4106 (0.806 to 20.931)	3746 (0.709 to 19.784)
Hypertriglyceridemia (not or yes)	6219 (1655 to 23.371) **	5420 (1395 to 21.049) *
Prenatal steroids administration ^a^ (not or yes)	1821 (0.496 to 6690)	1252 (0.332 to 4722)
Surfactant administration (not or yes)	2592 (0.629 to 10.686)	1799 (0.407 to 7959)
SGA at birth (not or yes)	0.510 (0.105 to 2487)	0.483 (0.095 to 2454)

**Notes.**^a^ Intramuscular steroid cycle in two doses of 12 mg over a 24-h period; GA (gestational age); ELBW (extremely low birth weight); FEF (full enteral feeding); SGA (small for gestational Age); * *p* value < 0.05; ** *p* value < 0.01. Data were expressed as mean (lower to upper limits 95% confidence interval).

## Data Availability

Data are available upon reasonable request. All data relevant to the study are included in the article. Access to raw data would be provided upon request.

## Supplementary Materials

The following are available online at https://www.mdpi.com/article/10.3390/nu13041243/s1, Table S1. Baseline clinical characteristic of newborns with gestational age ≤28 + 6/7 weeks or birth weight ≤1000 g.
