# Supplementary material for: Intravenous Lipid Emulsions Affect Respiratory Outcome in Preterm Newborn: A Case-Control Study
Source: Nutrients. 2021 Apr 9;13(4):1243. doi: 10.3390/nu13041243 (PMC8070203; doi:10.3390/nu13041243)
Supplement: Supplementary file 1 [file nutrients-13-01243-s001.pdf]

Table S1. Baseline clinical characteristic of newborns with Gestational Age ≤ 28 weeks or Birth Weight ≤ 1000 grams.

	**Cases of HiTG** *(n=34)*	**Controls** *(n=57)*
Maternal age, years	36 (32 to 39)	34 (32 to 36)
Gestational age, weeks	26 (25 to 27)	27 (26 to 28)
Birth weight, g	816 (678 to 954)	947 (867 to 1027)
SGA, No. (%)	14 (41.2)	26 (45.6)
Male sex, No. (%)	11 (32.4) **	6 (10.5)
Cesarean section, No. (%)	23 (67.6)	49 (86.0)
Intrauterine growth restriction, No (%)	5 (14.7)	4 (7.0)
Prenatal steroids administration [a], No. (%)	18 (52.9)	40 (70.2)
5-min Apgar score	7 (6 to 8)	7 (6 to 8)
pH at birth	7.2 (7.1 to 7.3)	7.2 (7.1 to 7.3)
Birth weight gain before 14 DOL, No. (%)	18 (52.9)	39 (68.4)
FEF, days after birth	37 (24 to 49) **	23 (18 to 27)
Fed with TPN (≥ 70%) in the first 7 DOL, No. (%)	25 (73.5)	47 (82.5)

Notes. (a) Intramuscular steroid cycle in two doses of 12 mg over a 24-hour period; HiTG (Hypertriglyceridemia); SGA (Small for Gestational Age); DOL (days of life); FEF (Full enteral feeding); TPN (Total parenteral nutrition). * vs Controls p value < 0.05; ** vs Controls p value < 0.01. Data were expressed as mean (lower to upper limits 95% confidence interval), when not specified.
